# Differences in the Communication of Cancer Diagnoses by Different Health Professionals and the Impact of Oncologist Communication on Patients’ Emotions

**DOI:** 10.3390/cancers16132444

**Published:** 2024-07-03

**Authors:** Elena Ruiz Sancho, Miguel Ángel Pérez Nieto, Francisco J. Román, Leticia León Mateos, Francisco Sánchez Escamilla, Santos Enrech Francés, María Ángeles Pérez Escutia, Ignacio Juez Mertel, Pedro Pérez-Segura, Andrea Aguirre Herrero, Marta Redondo Delgado

**Affiliations:** 1 HM Faculty of Health Sciences, Universidad Camilo Jose Cela, 28692 Madrid, Spain; mperez@ucjc.edu (M.Á.P.N.); lleon@ucjc.edu (L.L.M.); mredondo@ucjc.edu (M.R.D.); 2 Department of Biological and Health Psychology, Universidad Autónoma de Madrid, 28692 Madrid, Spain; f.javier.roman@uam.es; 3 Department of Medical Oncology, Hospital Universitario de Getafe, 28692 Madrid, Spain; 4 Department of Radiotherapy Oncology, Hospital Universitario 12 de Octubre, 28692 Madrid, Spain; 5 Department of Medical Oncology, Hospital Universitario de Fuenlabrada, 28692 Madrid, Spain; 6 Department of Medical Oncology and IdISSC, Hospital Universitario Clínico San Carlos, 28692 Madrid, Spain; 7 Researcher del Instituto de Psicología de Emoción y Salud, Institute of Psychology of Emotion and Health, 28692 Madrid, Spain

**Keywords:** anxiety, cancer, coping mechanisms, depression, oncology, physician–patient communications

## Abstract

**Simple Summary:**

Communication by health professionals impacts the mental health of cancer patients. This research sought to further explore this matter by studying the possible relationships between communication and a patient’s depression, anxiety, quality of life, coping strategies, and perception of their state of health. A total of 177 patients diagnosed with cancer answered a battery of questionnaires on these aspects. Our findings show that oncologists are better at delivering a cancer diagnosis than other healthcare professionals and that communication by them can impact patients’ mental and health variables. We believe that there is a need to implement better communication strategies among all healthcare professionals to facilitate the task of breaking bad news to patients. This will have a positive impact on patients’ emotional states and health while reducing stress and burnout among the healthcare professionals themselves.

**Abstract:**

The field of healthcare is increasingly adopting a humanistic perspective in the physician–patient relationship. One of the more salient aspects being studied is the communication between the two. This study serves a dual purpose. Our initial aim was to study how a cancer diagnosis is disclosed to patients by different physicians (GPs/other specialists/oncologists). Secondly, we set out to study how the way in which oncologists normally communicate with their patients impacts variables such as a patient’s anxiety, depression, coping mechanisms, and perception of both their health and their quality of life. A total of 177 patients answered a battery of questionnaires on sociodemographic and disease data: the SPIKES protocol, the EORTCQLQ-COMU26, and the ADAF screening questionnaire. The analyses recorded medium or high scores for some of the steps in the SPIKES protocol when delivering the diagnosis, and significant differences were observed for some of them among different physicians. The level of a cancer patient’s satisfaction with the communication by oncologists was related to their levels of anxiety, depression, vulnerability, and perception of their health and quality of life. Better communication strategies are called for among all healthcare professionals to facilitate the task of breaking bad news to their patients.

## 1. Introduction

Although the incidence of cancer is increasing [1], the diagnosis and treatment of cancer have both improved in recent decades. As a result, its associated mortality rate is falling [2]. However, the moment at which a patient is given their diagnosis and told about the treatment that they are going to receive constitutes a difficult process for the individual concerned and triggers a whole series of psychological consequences. Cancer patients are more likely than the general population to suffer from mental health issues [3,4,5]. 

These circumstances mean that the treatment of this disease needs to be biopsychosocial. A biopsychosocial perspective involves considering the psychological symptoms, coping strategies, perception of quality of life, and social support of cancer patients. All these aspects are related to a patient being able to adjust better to their situation [6,7,8]. The field of healthcare is increasingly adopting a humanistic perspective in the physician–patient relationship [9]. From this perspective, medicine should address the care of the whole person and not only focus on treating the disease [10]. Patient-centered care is characterized by engagement, empathy, and alignment with a patient’s beliefs and values [11]. Furthermore, in recent years, one of the more salient aspects that has been closely studied in this field is the communication between a patient and their physician. Different studies have confirmed the impact that a physician’s communication may have on a patient’s subsequent symptoms of depression and anxiety, and it may also influence their therapy options [12]. The evidence shows that patients who have been properly informed about their illness tend to adhere more to their treatment and show better long-term psychological adjustment [13]. It has also been reported that patients who feel very satisfied with the information received are better at adjusting to their circumstances and helping to improve the quality of their lives [14]. This research sought to further explore this matter by studying the possible relationship between communication and previously studied variables, such as depression, anxiety, and quality of life, while also investigating the possible influence that communication may have on the coping strategies a patient applies and their perception of their state of health. 

Doctors often have to deliver bad news to their patients [15]. A study by Baile et al. [16] presented data from a survey conducted by the American Society of Clinical Oncology (ASCO) in which around 500 oncologists answered questions on aspects related to how and when they broke bad news to their patients. It was revealed that 60% had to deal with the task of delivering bad news between five and twenty times per month, and as many as 15% of them had to do so more than twenty times over the same length of time. In spite of this rate, physicians receive little training in communication and the handling of emotions, with fewer than 10% of the surveyed oncologists receiving some kind of formal instruction for breaking bad news [16,17]. This means that oncologists are often overwhelmed and drained by these tasks, experience little confidence in their communication skills, and face such tasks with a degree of dread. This situation becomes even more stressful when the physician is inexperienced, the patient is young, or the outlook for the treatment’s success is unfavorable [18,19,20]. 

There are tools to help with this communication, such as the SPIKES protocol, which is used in many countries as the cornerstone for training courses [16]. The protocol structures a method for delivering critical news in six consecutive steps. The acronym SPIKES corresponds to the following dimensions: *Setting*—ensure a quiet environment and sufficient time; *Perception*—assess what the patient knows about the news and the expectations they have regarding the outcome that will be communicated; *Invitation*—explore the amount of information that the patient wants to be provided with; *Knowledge*—inform the patient about the diagnosis, prognosis, and health implications in understandable language; *Emotions and Empathic Response*—understand, validate, and empathize with the emotional reactions presented by the individual; and *Strategy and Summary*—communicate the plan, ask if it is understood, and encourage shared decision making. 

Although some patient preferences are not reflected in the steps of this protocol, it is the one most commonly used in physician training [21] and it has optimized the way in which bad news is delivered [22]. This protocol has been well received by health professionals, who confirmed in the study by Baile et al. [16] (2000) that they found it practical and easy to understand. Following the publication of the protocol, its application has subsequently been studied on several occasions in both cancer patients and patients receiving other types of life-changing diagnoses. However, the diverse use of this protocol by different healthcare professionals has barely been studied, and it has been even less studied in the case of cancer [23,24,25]. Sometimes, other specialists (gynecologists, pulmonologists, etc.) or GPs are the ones who have to diagnose cancer in a patient [26]. Because of this, this study evaluated and compared patients’ perceptions of the use of the SPIKES protocol among different professionals who informed them of their cancer diagnosis. 

Therefore, we pursued two goals: (1) to explore patients’ perceptions of the degree of compliance with practices in the communication of their diagnoses by evaluating their healthcare professionals’ adherence to the SPIKES protocol and comparing its use by different physicians (oncologists, other specialists, and GPs); and (2) to study whether the way in which oncologists interact with their patients impacts patients’ psychological variables, such as anxiety, depression, coping methods, and perception of health and quality of life. By achieving these objectives, we expect to be able to contribute information to the field of bad news communication in medicine, which has already been extensively studied. For the first objective, we provided a comparison of the fulfillment of the different steps of the SPIKES protocol among different health professionals. We hypothesized that there would be differences between them. For the second objective, we aimed to study the impact that a patient’s perceived satisfaction in the communication with their oncologist may have on variables already studied, including depression, anxiety, and quality of life, as well as the influence on the coping strategies used by oncology patients. In relation to this second objective, we expected that the level of a patient’s satisfaction in communication with their oncologist would have an impact on all the variables studied.

## 2. Materials and Methods

### 2.1. Participants and Procedure

Potential participants diagnosed with cancer were invited through the “Spanish Patient Forum” to contribute to a study aiming to investigate and improve the way in which doctors deliver bad news and communicate with their patients. A total of 177 patients from three cancer associations responded to the questionnaires through an online form sent to them by e-mail. Data were gathered between September 2020 and March 2021. The inclusion criteria were patients who were aged over 18, diagnosed with cancer, receiving treatment or being monitored, and willing to sign an informed consent form (which was included as part of the online form) for taking part in the research. This study was approved by the Ethics and Clinical Research Committee of the Hospital Clínico San Carlos. This study was designed as a cross-sectional study. The disease prognosis was obtained from two consultant oncologists who considered the type of cancer and the time elapsed since the diagnosis was delivered. This information was used to analyze each one of the participants, and they were classified according to a curable or non-curable prognosis. 

### 2.2. Instruments

The sociodemographic variables were evaluated through an ad hoc questionnaire that included aspects related to the person’s illness as well as a self-assessment of their health and quality of life on a scale of 1 (very poor) to 7 (excellent). The items used to measure the latter two variables were selected from the EORTC quality-of-life questionnaire QLO-C30 (3.0) [27]. This questionnaire could not be completed in its entirety due to the associations’ requirement to ask a short battery of questions.

The evaluation of the disclosure of the diagnosis involved a Likert-type questionnaire with six items ranging from 0 (not at all) to 10 (a great deal) that assessed the degree of adherence to the SPIKES communication protocol [16]. We generated 6 Likert-scale survey questions. The reliability of the questionnaire in this sample was α = 0.910. The questions were drawn from the SPIKES framework and captured each of the six components. In addition, an instrument has been developed by another research group that measures patients’ compliance with this protocol [24]. It is a 17-item instrument (with most of the items having a dichotomous response format) that has not been validated in Spanish. For the elaboration of our 6 items, we based the use of this instrument and others generated by other research groups on objectives related to the SPIKES protocol. Mirza et al. [21] (2019) compared the steps of the SPIKES protocol with patients’ preferences, and in the studio of Abdalazim Dafallah et al. [28] (2020), the doctors themselves evaluated whether or not they had followed the steps of the SPIKES protocol. We wanted a brief instrument and were interested in a response format that assessed the degree of adherence in a quantitative, rather than dichotomous, manner. Therefore, in our study, the patients were asked whether the physician delivering the diagnosis (a) dedicated enough time to them; (b) made sure there was adequate privacy for holding their conversation; (c) checked whether the patient knew anything about the disease prior to disclosing the diagnosis; (d) checked what the patient wanted to know and what they considered most important prior to disclosing the diagnosis; (e) helped the patient to express and handle their feelings of sadness, fear, anxiety, and others; and (f) clearly explained the next steps to be taken to address the disease. 

The patients’ satisfaction with the communication was evaluated using the EORTC QLQ-COMU26 scale [29] drawn up by the quality-of-life group at the European Organisation for Research and Treatment of Cancer (EORTC) to assess patients’ satisfaction with their communication with their oncologist and the information received. This scale can be used for patients with any kind of cancer at any stage of the disease. It contains 26 items, with each one being scored on a Likert-type scale from 1 (not at all) to 4 (a lot). The instrument has adequate reliability for all scales (internal consistency > 0.70; test–retest reliability > 0.85) and demonstrates good convergent validity as a measure of patients’ perceptions of their communication with health professionals [30].

Negative emotions and coping strategies were evaluated with the ADAF screening questionnaire (ADAF is the Spanish acronym for Ansiedad, Depresión, AFrontamiento—Anxiety, Depression, and Coping). This instrument was developed by our research team for screening these issues in cancer patients. It has a two-dimensional structure: (a) negative emotions, with three items—one related to anxiety and two related to depressive symptoms—and (b) dysfunctional coping strategies, with two items—the first one measures helplessness and the second one measures avoidance. The items are scored on a four-point Likert-type scale from 0 (almost never) to 3 (almost always) and the instrument has suitable levels of reliability and good sensitivity (values between 67.2% and 83.3%) and specificity (values between 61.9% and 89.6%) [31].

### 2.3. Statistical Analyses

The first step involved calculating the descriptive statistics for this study’s sociodemographic variables (mean, standard deviation, and frequency distribution). The first of these objectives involved the instrument’s items that evaluated the use of the SPIKES protocol as quantitative variables on a scale of 1–10. Group comparisons were made between the various healthcare physicians (GPs/other specialists/oncologists) using the Kruskal–Wallis H test due to differences in the participants’ distribution in each group (GPs = 12, oncologists = 20, and other specialists = 145). The effect size was computed using the epsilon-squared (ε^2^) statistic designed for the Kruskal–Wallis test; ε^2^ depicts the variance explained by group differences. The Bonferroni correction was applied for any significant differences.

The second step calculated the mean descriptors and standard deviation for the following variables: the ADAF, the EORTC QLQ-COMU26, and the perception of health and quality of life. This perception and the various psychological variables were predicted using multiple regression analysis with the following predictor variables: sex, educational level, socioeconomic status, occupation, the prognosis for the disease, time elapsed since the diagnosis, and overall score in the perceived satisfaction with the physician–patient communication. We analyzed the presence of multicollinearity among the variables (tolerance > 0.10 [32] (Pituch and Stevens, 2016) and the variance inflation factor (VIF) < 5 [33]. The values were appropriate, suggesting that there were no issues of multicollinearity. The independence of errors was checked using the Durbin–Watson statistic with values between 1.5 and 2.5 for all regression models [34].

## 3. Results

Table 1 shows the sociodemographic data of the sample and the data related to each patient’s disease. The mean age of the sample was 52.07 years, with a range from 21 to 76. The majority of the participants were women (79.1%). The most frequent types of cancer were lung (30.51%) and ovarian (28.25%). The diagnosis of cancer was reported mainly by other specialists (80.56%), mostly gynecologists, pulmonologists, and digestive physicians.

Table 2 shows the descriptors for compliance with the SPIKES protocol by the different types of physicians and the differences between them in the Kruskal–Wallis H test.

The items with the lowest overall scores were those involving the questions on what the patients wanted to know before receiving the diagnosis, on their prior understanding of it, and on the expression and control of emotions. The highest-scoring items involved the privacy of the setting where the diagnosis was delivered and sufficient time taken. The comparisons between the categories of physicians revealed significant differences in the overall scores for the protocol, the evaluation of the patient’s prior understanding of the disease, and the evaluation of the aspects that the patient wanted to know about and considered most important, with oncologists receiving the highest scores. 

With regard to the second objective, Table 3 presents the descriptors for the variables. The cut-off point for suspecting problems of anxiety and inadequate use of coping strategies was 2, and it was 3 in the case of depression [31]. Our study found that all the scores were below this threshold. With regards to the level of perceived satisfaction of the patients, the items in the questionnaire were added up and the obtained mean was 72.96. The perception that the patients had of their state of health was 5.08, and it was 5.16 for their quality of life.

Continuing with the second objective, Table 4 presents the variables that may predict a patient’s levels of depression and anxiety, coping mechanisms, lifestyle health, and quality of life. A patient’s satisfaction with their physician–patient communication predicted all the dependent variables except for the avoidance coping strategy. The figures for explaining these models varied between 2.5% and 15.1%. 

## 4. Discussion

This study aimed to explore various aspects of communication between health professionals and oncology patients. We contributed the following main findings: (1) Firstly, data were obtained on patients’ perceptions of how well the SPIKES protocol was followed by the health professional who diagnosed them with cancer. This highlighted that health professionals, in general, show more adherence to some steps of the protocol than others. (2) The follow-up of this protocol by the different health professionals (oncologists/other specialists/GPs) was compared and interesting findings were found, reflecting that oncologists adhere more closely to some of the steps of the protocol than other healthcare professionals. (3) Finally, it was determined that patients’ perceived satisfaction with regular communication with their oncologist had an impact on relevant variables, such as patients’ emotional states or their perception of their quality of life. All of these findings are discussed in more detail below. 

### 4.1. Patients’ Perception of SPIKES Protocol Follow-Up 

With regard to the first objective, sufficient time was devoted to the measures taken to ensure privacy and explain the next steps to be taken. However, the items related to the questions before the diagnosis was delivered and the help provided to the patients when expressing and controlling their emotions recorded lower scores. In our opinion, these three steps may be related. The emotional impact on a patient receiving a cancer diagnosis is directly related to the gap between their perception of what is happening to them and the information received. This makes it difficult to adopt an empathetic approach to patients’ emotional reactions if we do not already understand their expectations of the information they are about to receive. These findings coincide with those of other studies. The aforementioned study by Baile [16] states that the most challenging stages of the SPIKES protocol are the *Emotions and Empathic Response*, *Perception*, and *Invitation* stages. From the patients’ perspectives, the findings were similar. Cancer patients perceived that they were not asked about the information they wanted to receive before being given their diagnosis or what they would have considered most important [35]. The steps of *Perception*, *Invitation*, and *Strategy and Summary* also received low scores in the study by Marschollek [24], in which a generally low level of satisfaction with the information received was recorded for the patients. 

### 4.2. Comparison between Different Health Professionals

One of this article’s contributions is a comparison of the fulfillment of the SPIKES protocol steps by various categories of physicians. It may, therefore, be noted that the two items with the lowest scores, namely the questions prior to receiving the diagnosis, received significantly lower scores among other specialists than among oncologists. These significant differences also appeared in the overall score recorded for the fulfillment of the protocol. These data show that other specialists are probably less adept at performing these tasks than oncologists with greater experience in breaking bad news. Although none of them received the necessary training, the everyday experience of oncologists may enable them to improve certain aspects of their communications, whereas other specialists are not presented with as many opportunities to do so. This may seem somewhat paradoxical, considering that other specialists are often the ones who have to deal with the initial cancer diagnosis. This communication of a cancer diagnosis by medical specialists probably happens frequently, but it occurs in isolation from the different tasks they perform on a daily basis. For example, a gynecologist will attend regular check-ups for women, pregnancy controls, etc. Only on specific occasions will a gynecologist need to communicate a diagnosis of cancer. However, it is common for oncologists to regularly talk about diseases that cause a lot of discomfort and that can have fluctuations in their treatment. This clearly highlights the need for proper training. These differences were not found for other steps in the protocol, such as devoting enough time, ensuring privacy, helping to express and control emotions, and explaining the next steps to be taken. It appears that certain aspects, such as the setting and the time involved, were handled well by all the physicians, and different studies have identified these aspects as being very important for patients [36]. We did not find any other studies that have compared different healthcare professionals, but the need for training has been highlighted in other studies that have evaluated nursing staff or GPs [37,38]. The difficulty that healthcare professionals may perceive in communicating a diagnosis of cancer is highlighted when, at times, they prefer not to communicate it or provide the information clearly. Some studies have identified that, sometimes, other specialists prefer not to make a diagnosis and will instead wait for the oncologist to do so [39]. This is a problem for the health field. Studies have reported that, when a patient is aware of their diagnosis, there may be an increased sense of control and they may connect more with the physician informing them [40]. Additionally, it is not only patients who are affected by the communication of a diagnosis. In cases where practitioners present the diagnosis and openly discuss the possible complications that may occur during the necessary interventions, the possibility of medical malpractice is reduced [41]. The professional responsibility in the management of a disease such as cancer does not belong to one person alone. Behind every procedure, there is a multidisciplinary team that works together to provide comprehensive treatment. Therefore, clear communication about the diagnostic process and treatment is a fundamental task among the different professionals involved [42].

It is interesting to note the low score recorded for the item “*The physician helped me to express and handle my emotions: sadness, anger, fear, anxiety or others*”. No significant differences were found for this step, yet all the scores were low across the board for oncologists, GPs, and other specialists. This seems to be an aspect that needs to be improved in physician–patient communications, regardless of the category of healthcare professionals being evaluated. Oncologists themselves have already singled out these shortcomings in previous studies [20]. In turn, cancer patients have expressed the importance they place on the emotional support provided by their physicians at all stages of the disease [43]. 

### 4.3. Relationship between Perceived Satisfaction in Communication with the Oncologist and Study Variables

With regards to the second study objective, none of the variables measured with the ADAF scored more than the cut-off point, and thus, the risk of anxiety, depression, and the use of concerning coping mechanisms was considered low. High scores were recorded for the perception of health and quality of life, as well as for the satisfaction perceived by patients in their communication with their oncologist. With regards to this last aspect, the results reported in different studies are contradictory: in some studies, patients expressed their overall satisfaction with their communication with physicians, while the perceived satisfaction has been low in other cases [44,45]. 

Nevertheless, our study highlights that physician–patient communication impacted the studied variables. The patients’ perceived satisfaction with the communication was related to all the variables studied except for the avoidance coping strategy. Better communication was associated with lower levels of depression, anxiety, and vulnerability, and with higher levels of the perceived health and quality of life. These findings are consistent with those reported in other studies [29,38,46,47,48]. Our study also found other variables that are predictive. A good prognosis was linked to lower levels of depression and a better perception of health and quality of life. A high educational level was related to the reduced use of an avoidance coping strategy, while a low socioeconomic status resulted in higher levels of depression. Other studies have also stressed the importance of the stage of a patient’s disease and how this is related to their coping strategies and emotional state [49,50]. Further analysis of these last variables revealed that the prognosis, educational level, and socioeconomic status are more static variables, over which a physician does not have very much control or leeway to make major changes. This again highlights the need to invest time and resources into teaching our healthcare professionals. This is more important now, after the COVID-19 pandemic, because it has been shown that cancer patients experience higher levels of depression and anxiety [51]. Providing training to health professionals in breaking bad news has a clear impact on improving the performance of health professionals and on patients’ perceptions of the performance, without the need to arrange an excessive number of teaching sessions [52,53]. This leads to higher levels of self-confidence among physicians and less stress when dealing with this task [13]. Other studies have already focused on this matter, suggesting that this instruction should be included as part of a degree in medicine and continue throughout post-graduate courses and professional careers [31]. The data indicate that not all patients have the same preferences when talking to their physicians, and there is a need for health professionals to adapt to the different characteristics of their patients (age, sex, stage of the disease, educational level, etc.). The training of healthcare professionals also needs to include these aspects [53,54].

### 4.4. Study Limitations

This work has several limitations. Firstly, in relation to the selection of the evaluation instruments, the instrument used to measure the follow-up of the SPIKES protocol was generated by the research group itself. We intend to validate this in future research. The variable *perception of health and quality of life* was measured using a single item, despite the existence of validated instruments for this assessment, such as the QLO-C30 (3.0) [27]. The reason for using the evaluation format of this study was to accommodate a request from the involved associations to shorten our battery of instruments. The data were gathered online because this research coincided with the COVID-19 pandemic. This may have influenced the emotional state of the patients and the communication they had with their oncologists due to the use of masks. Nonverbal communication may have been affected by this. Finally, we used a sample of patients who received their diagnosis a long time ago. This may have had an influence on various variables and on patients’ perceptions of their communication with their oncologists, whom they had been dealing with for a long time. It would be expedient to extend this study by exploring differences according to the time elapsed since the diagnosis and other variables, such as the current stage of the cancer. 

## 5. Conclusions

Our findings show that oncologists are better than other healthcare professionals at some of the steps in the SPIKES protocol for communicating a cancer diagnosis. In addition, the communication between a patient and a health professional can predict the mental and health variables of patients. We believe there is a need to implement better communication strategies among all healthcare professionals to enable them to better handle the task of breaking bad news to patients. This would have a positive impact on patients’ emotional states and health while reducing stress and burnout among the healthcare professionals themselves.

## Figures and Tables

**Table 1:** Demographic and clinical characteristics of the participants (*N* = 177).

Variable (Range)			*N*	%
Age (21–76)		Mean (SD)	52.07 (10.17)	
Sex	Male		37	20.9
	Female		140	79.1
Nationality	Spanish		174	98.3
	Other		3	1.7
Civil status	Married/registered partnership		135	76.3
	Separated/divorced		18	10.2
	Single		20	11.3
	Widowed		4	2.3
Educational level	No studies		1	0.6
	Primary–secondary		54	30.5
	Tertiary		122	68.9
Occupation	Homemaker		9	5.1
	Unemployed		15	8.5
	Student		2	1.1
	Retired		18	10.2
	Retired through illness or disability		55	31.1
	Active employment		62	35.0
	Other		16	9.0
Socioeconomic status	Low		6	3.4
	Medium–low		76	42.9
	Medium–high		93	52.5
	High		2	1.1
Type of cancer	Digestive		29	16.38
	Lymphoma		2	1.12
	Breast		28	15.81
	Ovarian		50	28.25
	Lung		54	30.51
	Uterus		8	4.52
	Uterus and colon		2	1.12
	Bladder		4	2.26
Prognosis	Curable or oncology prognosis		105	59.3
	Non-curable oncology prognosis		72	40.7
Healthcare professional who provided the diagnosis	GP		12	6.67
	Oncologist		20	11.11
	Other specialist		145	80.56
Mean time elapsed since the diagnosis in months (1–192)		Mean (SD)	47.65 (39.09)	
	Less than one year		30	16.7
	Between 1 and 2 years		29	16.1
	Between 2 and 3 years		38	21.1
	Between 3 and 4 years		17	9.4
	Between 4 and 5 years		26	14.4
	More than five years		40	22.2

SD: standard deviation.

**Table 2:** Results and comparison of compliance with the SPIKES protocol among physicians.

Variable	Total		Oncologists (20)		GPs (12)		Other Specialists (145)		H (2); *p*	E^2^ R	Post Hoc Bonferroni
	Mean	SD	Mean	SD	Mean	SD	Mean	SD			
Sufficient time	7.25	2.78	8.45	1.39	7.17	2.88	7.09	2.89	2.94; 0.229	0.016	
Privacy	7.48	2.93	8.45	1.70	7.75	3.33	7.32	3.02	1.79; 0.407	0.011	
Inquired about my prior understanding of the disease	4.75	3.66	7.15	2.94	5.25	3.59	4.38	3.64	10.89; 0.004 *	0.062	Oncologists > other specialists 0.003
Before breaking the news, the physician asked what I wanted to know	4.33	3.61	6.40	3.5	5.00	3.16	3.99	3.58	8.79; 0.012 *	0.050	Oncologists > other specialists 0.011
The physician helped me to express and control my emotions	4.83	3.65	5.95	3.45	5.92	3.23	4.59	3.68	3.55; 0.169	0.020	
Explanation of the next steps	6.75	3.37	8.20	2.09	7.58	3.55	6.48	3.45	5.14; 0.076	0.029	
Total for the questionnaire	35.39	16.74	44.60	13.34	38.67	17.16	33.85	16.78	7.95; 0.019 *	0.045	Oncologists > other specialists 0.019

SD: standard deviation; H: Kruskal–Wallis; * *p* ≤ 0.05.

**Table 3:** Descriptive statistics of the study variables.

Variable (Range)	Mean (SD)
ADAF	
Anxiety (0–3)	1.58 (0.85)
Depression (0–6)	2.54 (1.68)
Helplessness (0–3)	0.91 (0.93)
Avoidance (0–3)	1.66 (0.87)
EORTC QLQ-COMU26 Satisfaction with physician–patient communication (0–104)	72.96 (16.74)
Perception of health (1–7)	5.08 (1.30)
Quality of life (1–7)	5.16 (1.34)

SD: standard deviation.

**Table 4:** Linear regression analysis.

Dependent Variables	Significant Predictors	β	*p*	Adjusted R^2^
Anxiety ^1^	Satisfaction with physician–patient communication	−0.23	0.002	0.049
Depression ^2^	Satisfaction with physician–patient communication	−0.18	0.012	0.142
	Prognosis	−0.23	<0.011	
	Socioeconomic status	−0.20	0.006	
Helplessness ^3^	Satisfaction with physician–patient communication	−0.30	<0.001	0.084
Avoidance ^4^	Educational level	−0.17	0.019	0.025
Perception of health ^5^	Satisfaction with physician–patient communication	0.29	<0.001	0.121
	Prognosis	0.19	0.009	
Perception of quality of life ^6^	Satisfaction with physician–patient communication	0.30	<0.001	0.130
	Prognosis	0.19	0.007	

β: beta/standardized coefficients. ^1^ Durbin–Watson = 1.649, tolerance = 1.000, and VIF = 1.000; ^2^ Durbin–Watson = 2.024, satisfaction with physician–patient communication (tolerance = 0.924; VIF = 1.082), prognosis (tolerance = 0.982; VIF = 1.018), and socioeconomic status (tolerance = 0.939; VIF = 1.065); ^3^ Durbin–Watson = 1.641, tolerance = 1.000, and VIF = 1.000; ^4^ Durbin–Watson = 1.740, tolerance = 1.000, and VIF = 1.000; ^5^ Durbin–Watson = 1.945, satisfaction with physician–patient communication (tolerance = 0.984; VIF = 1.016), and prognosis (tolerance = 0.984; VIF = 1.016); and ^6^ Durbin–Watson = 1.857, satisfaction with physician–patient communication (tolerance = 0.984; VIF = 1.016), and prognosis (tolerance = 0.984; VIF = 1.016).

## Data Availability

The data that support the findings of this study are available from the corresponding author upon reasonable request.
